# Cellular demiurgs – engineer to understand

**DOI:** 10.1007/s00709-026-02198-7

**Published:** 2026-04-15

**Authors:** Peter Nick

**Affiliations:** Joseph Gottlieb Kölreuter Institute für Plant Sciences, Karlsruhe Institute of Technology, Karlsruhe, Germany

In an attempt to explain the origin of the universe, Plato, in his *Timaios* dialogue (written around 360 BC, for an English translation see Burnet 1902), introduced a Heavenly Craftsman, Demiurgos, who, by the power of his intellect, molded the chaotic and lawless matter into a cosmic order, governed by the Laws of Nature. He built the universe according to a perfect, beautiful, and eternal model. For doing so, he had to overcome the resistance of the already existing matter by “persuading” its necessities, striving to create a world that is good. The demiurgs of biology are driven by two motivations: In the first place, engineering can reshape living organisms to fulfil human needs. However, engineering can also serve epistemological purposes. Only, when they are able to dismantle and re-assemble them, engineers can claim to have fully understood their machines. Three contributions to the current issue illustrate different demiurgic approaches to living organisms with the purpose to extract knowledge from them.

The study by Liu et al. (2026) describes, how a combination of molecular engineering and cutting-edge imaging equipment can help to dissect protein-protein interactions in living plants. Signal transduction often uses multi-component complexes that can be re-assembled in a dynamic and modular fashion in response to environmental signals including stress signals. To what extent the components in such a complex are entertaining physical interaction, and to what extent they are just localised in similar sites of the cell, cannot be resolved by light microscopy, because the resolution limit (around 250 nm) gives ample space to accommodate up to ten intermediate proteins between two partners imaged, for instance, by fluorescent probes. Biochemical approaches, like yeast-2 H, pull-down assays, or proximity-labelling strategies, can demonstrate direct physical interaction, but strip their candidate from their cellular context and, therefore, suffer from excessive reductionism. The fact that Fluorescence Resonance Transfer decreases with the 6th power of distance between two fluorescent molecules, has been used to demonstrate proximity of 5–10 nm, used as indication for physical interaction, and the power of this method can be even increased by integrating Fluorescence-Lifetime Imaging (Bücherl et al. 2014). However, this approach not only requires sophisticated instruments, which is expensive, but also substantial expertise to avoid mis-interpretations. A more user-friendly strategy has been Bimolecular Fluorescence Complementation (Stolpe et al. 2005), where the two binding partners are fused to complementary halves of the Yellow Fluorescent Protein, such that upon interaction, a fluorescent signal is generated. A drawback of this robust method is the irreversibility of the fluorophore re-constitution, which will mask the often transient nature of the interactions within a signalling complex. The authors go for a similar approach, but instead of fluorescence, they utilise the more dynamic bioluminescence of a split-luciferase system to dissect the interactions in a complex mediating the effect of blue light upon hormonal signalling. The underlying complex belongs to the family of the Cullin4-E3 ubiquitin ligases, central players in plant signal transduction. This complex can entertain dynamic interactions with the blue-light photoreceptor cryptochrome on the one hand, and with PYR-like 8, a receptor for the plant hormone abscisic acid that is a master regulator for adaptation, mainly in the context of drought and cold stress. While such split-luciferase have been used already to probe for protein-protein interactions *in planta*, the novelty of the current work is to quantify the strength of the interactions, which is highly relevant in signalling, where the binding partners contact only transiently and often not very tightly. A pre-condition is very efficient imaging, which is provided here by the nvoel NightShade bioluminescent system. Using transient transfection of tobacco leaves as host, the authors give a proof-of-principle by demonstrating a interaction of the abscisic-acid receptor with the complex component DDA1. This interaction holds valid against a series of negative controls demponstrating its specificity and robustness. Overall, the combination of biological and technical engineering can help to probe protein-protein interactions in planta, opening new strategies to map dynamic interactions during signalling.

Also, the work by Shkryl et al. (2026) in the current issue represents a proof-of-concept for a new strategy of plant engineering. They combine artificial micro-RNAs and extracellular vesicles to silence GFP as traceable target. Extracellular vesicles derive from multivesicular bodies that fuse with the plasma membrane. Originally discovered in animal cells, they were later also found in plants (Marcote et al. 2000) and play a role during defence against pathogens. While conventional exocytotic vesicles will dilute their content into the apoplast, extracellular vesicles sequester their content from diffusion and, due to their surface properties, they can fuse with specific target membranes, delivering their cargo in a compact manner, modulating the signalling of the recipient cell (which in case of defence would be the attacking pathogen). For instance, plants can use extracellular vesicles to target specific microRNAs directed against pathogen virulence factors, such that the transcripts for these factors are then recruited to the miRNA-induced silencing complex and degraded. The authors work with artificial microRNAs inducing silencing of a GFP reporter. They isolate extracellular vesicles using a differential ultracentrifugation protocol from either non-transformed wildtype plants or from plants expressing these constructed microRNAs. The size of these vesicles, determined by a nanoparticle tracker and by Scanning Electron Microscopy, is in the range of about 100 nm. When they administer these vesicles to either protoplasts or leaves from GFP-expressing plants as recipients, they observe a significant decline in the fluorescent signal, faster in the protoplast system, somewhat slower in the plant recipient. This is indicative of successful silencing, which is confirmed by transcript measurements for one of the microRNAs. Since this proof-of-concept also works for leaves as recipient, albeit slower than in protoplasts, the method could be used in the future to get transient silencing in plants without the need to go through the often cumbersome or even recalcitrant transformation process.

The third study highlighted here, by Harada et al. (2026) also uses cellular engineering, but already moves a step beyond a proof-of-concept. They are focus on mastigonemes, small appendices at the flagellum of Stramenopiles. As shown by the late Cavallier-Smith (2022) in his final legacy published in this journal, details of the flagellum can serve to tame the overboarding diversity of basal eukaryotes into a convincing phylogeny. Also the Stramenopiles, as seemingly heterogenous a group of life forms comprising diatoms, brown algae, but also oomycetes can be aligned due to the fact that they share two flagella that are inserted laterally and pull the cell forward. In contrast, the Opisthokonts, comprising animals and true fungi, group together, because their sperm cells are flagellated at the rear thrusting the cell forward. The authors revisited the hypothesis that this thrust reversal of the flagellum is linked with the presence or absence of mastigonemes (Cahill et al. 1996). As experimental model, they use gametes of the brown alga *Ectocarpus*. In the oomycete *Phytophthora parasitica*, a combination of immunofluorescence and GFP-tagging had identified the protein MAS1 as main component of the mastigonemal shaft (Hee et al. 2019). In their previous work, the authors had established a method for targeted knock-out in their organism using micro-injection of CRISPR-Cas9 ribonucleotide and a 2-fluoroadenine resistance as selection marker (Badis et al. 2021). Using this strategy, the authors succeeded to engineer a *mas1* loss-of-function mutant. In fact, the gametes of this mutant were void of any mastigonemes as demonstrated by immunofluorescence and Transmission Electron Microscopy. Likewise, the mutant gametes were swimming much slower than the wild type, but increased the frequency of their beats. This finding supports a role of mastigonemes for the effeciency of the flagellar thrust. To address potential roles for MAS1 on side of the oocyte, the authors established a female mutant strain, but the mutant cells were normally fertilised, independently, whether mutant or wild-type sperm were used. Thus, MAS1 is relevant for the flagellar thrust, but dispensable for gamete recognition and fusion.

There has been an endless debate on the possible motivation that drove Plato’s Demiurgos to engineer the world into a cosmic order. Most philosophers argue that it was a sense of aesthetics to tailor the real world to align with an ultimately geometrical plan. This would be clearly a project of engineering. In contrast, Richard Mohr (1983), in a famous essay, pointed out that Demiurgos acted out of an epistemological strive. Demiurgos engineers in order to understand. Whether this philosophical debate can be decided, has to remain open. However, it is evident that the cellular demiurgs of our time use engineering to gain knowledge, in the first place.
